# Concentration-Dependent Nonlinear Rheology of Agar Hydrogels

**DOI:** 10.3390/gels12070603

**Published:** 2026-07-07

**Authors:** Marko Volk, David Stopar

**Affiliations:** Department of Microbiology, Biotechnical Faculty, University of Ljubljana, Večna pot 111, 1000 Ljubljana, Slovenia; marko.volk@bf.uni-lj.si

**Keywords:** agar, nonlinear rheology, LAOS, energy dissipation, strain stiffening, shear thickening

## Abstract

Despite decades of research, the nonlinear mechanics of agar remains poorly understood. In this work, we analyze the mechanical response of soft, hard, and very hard agar hydrogels under nonlinear shear deformation. Low-shear viscoelastic behavior across concentrations was characterized using storage and loss moduli, yield strain, flow point, loss factor, and ductility index. The transition to nonlinear response was examined using Fourier analysis of shear stress signals. To describe the high-shear regime, we employed large-amplitude oscillatory shear (LAOS) rheology. The mechanical response was further analyzed using Lissajous–Bowditch plots (stress versus strain and stress versus shear rate), linking agar network structure to intracycle deformation behavior and energy dissipation. By analyzing strain stiffening, shear thickening, yielding, and intracycle structural dynamics, we quantified dissipation rates across concentrations and constructed fingerprint maps of shear stiffening and thickening at different frequencies. Microstructural insights from rheology were compared with macroscopic characterization using phase-contrast microscopy. The nonlinear rheological analysis revealed that structural reorganization shifts systematically toward lower shear strain values with increasing agar concentration.

## 1. Introduction

Among the gelling biomolecular hydrocolloids commonly used in industry, agar occupies a distinctive position due to its high gelling efficiency and pronounced thermal hysteresis [1]. Agar is extracted from red algae and consists primarily of two polysaccharide fractions: agarose, the neutral gelling component, and agaropectin, a more heterogeneous, and partially charged fraction [2,3,4,5]. When heated in aqueous solution, agarose chains dissolve and adopt a random coil conformation. Upon cooling, gelation typically occurs between 43 and 32 °C. On the other hand, melting requires significantly higher temperatures (approximately 85–95 °C), resulting in pronounced thermal hysteresis [6,7,8]. At the molecular level, gelation proceeds through a two-step mechanism: individual chains transition from random coils to ordered double helices through intermolecular hydrogen bonding, followed by aggregation of double helices into junction zones that form the physical cross-links of a thermoreversible three-dimensional gel network [9].

The concentration of agar has a pronounced effect on the architecture of the gel network. At low concentrations, chain density is insufficient to support extensive helix bundling, and the resulting network is sparse, with large mesh sizes and few junction zones. As concentration increases, helix aggregation becomes more extensive, producing denser, more interconnected networks with smaller mesh sizes and a higher cross-link density. At low concentrations, agarose gels are soft, while at high concentrations they become markedly more rigid, with elasticity arising from thick, connected bundles that form the junction zones of the network [5]. The structural differences have been directly visualized using microscopy techniques. Atomic force microscopy (AFM) in aqueous conditions has shown that pore diameter decreases as agarose concentration increases, and that the pore size distribution narrows as gel concentration increases [10]. Complementary imaging by confocal laser scanning microscopy (CLSM) has revealed that the network structure densifies with increasing agarose concentration, transitioning from loosely connected fibrillar arrangements to compact, highly bundled architectures [11]. Cryo-TEM, AFM, and SEM have together revealed that agarose forms shape-persistent helical fibrils of approximately 10 nm diameter that bundle into semiflexible fibrillar networks whose morphology is qualitatively similar across a range of concentrations [10,12].

Agar behaves predominantly as elastic material [5,13]. Under small deformations, e.g., by gentle pressing with the tongue, agar is perceived as a firm, springy bite with smooth surface coating in mouth, which determines the initial mouthfeel. Soft agar desserts with lower concentration feel like they melt in the mouth, whereas stronger gels feel more solid but still smooth. With larger deformations under chewing or large bite forces, agar exhibits brittle fracture with cracks instead of deforming plastically. Sudden structure collapse once yield stress is exceeded feels like a crisp or brittle bite in firm agar gels. Agar gels lack chewiness compared to gelatin, which at high agar concentration gives an unpleasant, brittle mouthfeel [14,15]. The mechanical properties of agar are well captured by oscillatory amplitude shear measurements in the linear viscoelastic region. It has been shown that with increasing agarose concentration the elastic modulus of the gel increases, consistent with the theory of cross-linked purely elastic gels [11,13]. The elastic modulus has been shown to follow a power-law dependence on concentration close to an exponent of 2, consistent with network formation through double-helix cross-linking [16]. When subjected to large shear deformations that exceed the linear viscoelastic regime, the network undergoes progressive structural disruption, and material behavior is governed by nonlinear mechanics rather than equilibrium elastic properties. For analyzing the nonlinear stress–strain response in complex material, large-amplitude oscillatory shear (LAOS) rheology has become an increasingly popular analytical tool [17,18,19,20,21]. LAOS provides insight into phenomena such as yielding, strain stiffening or softening, shear thickening, and structural breakdown, making it particularly valuable for complex systems like gels, biofilms, and food materials [22]. The literature on polysaccharide gel LAOS behavior is extensive [7,13,16,23,24,25,26,27,28]. However, compared with alginate, gelatin, and CMC systems, LAOS studies of agar remain relatively limited. In particular, systematic investigations of concentration-dependent strain-amplitude sweeps are scarce, and comprehensive analyses based on higher harmonics and intracycle decomposition of the elastic and viscous responses are largely absent from the literature.

Here we analyze linear and nonlinear rheological response of agar gels across a range of concentrations. Agar concentrations were selected in a geometric progression (0.75, 1.5, 3, and 6%), with each concentration doubled relative to the previous one, to generate gels spanning a broad range of mechanical properties from soft to very stiff. Storage and loss moduli, yield strain, flow point, loss factor, and ductility index were used to characterize linear viscoelastic behavior of agar across different concentrations. To characterize the transition to nonlinear response Fourier analysis of the shear stress response curves was used. The nonlinear agar responses at large deformations were analyzed with LAOS rheology. The degree and nature of nonlinearity were evaluated by Lissajous–Bowditch plots (stress versus strain and stress versus shear rate), which provide a direct visual representation of deviations from linear viscoelastic behavior, revealing features such as strain stiffening or softening, shear thickening yielding, and intracycle structural changes [21]. We determined the dissipation rate across agar concentrations and made fingerprint maps of strain stiffening and shear thickening at different frequencies. Together, these approaches enable a detailed characterization of the agar’s nonlinear viscoelastic properties under large deformation that provides a comprehensive overview of agar mechanical behavior under stress.

## 2. Results

### 2.1. Microscopy Imaging

**Figure 1:**
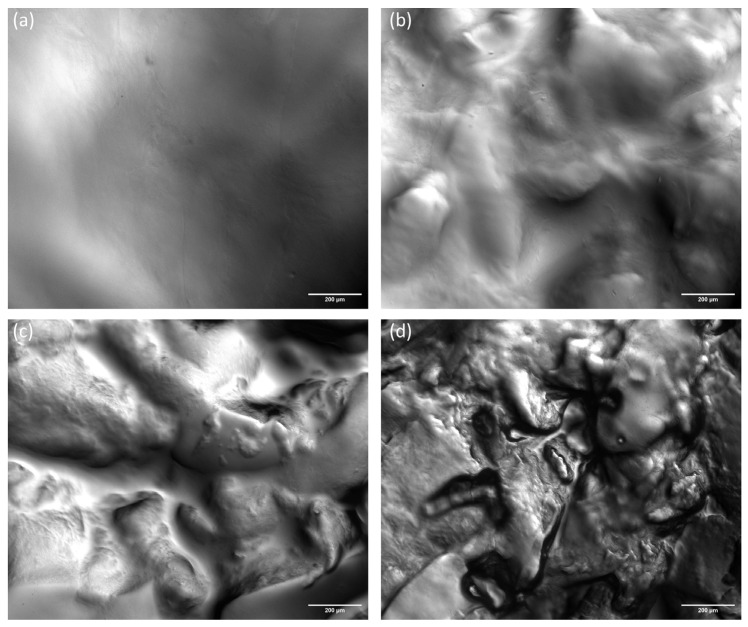
Microscopic images of agar surface gentle scraped to induce fracture at different concentrations: (**a**) 0.75, (**b**) 1.5, (**c**) 3, and (**d**) 6% agar.

Micrographs of agar surface morphologies at different agar concentrations that were mechanically disrupted by gentle scraping to induce fracture are shown in Figure 1. At 0.75% agar, the surface appears even and smooth. With increasing agar concentration, the surface becomes progressively rougher on the micrometer scale and fractures remain visible. At the highest concentration (6%), the surface was clearly textured, exhibiting coarse, irregular features on the order of ~200 µm. This is consistent with a stiffer, more brittle gel network at higher polymer concentrations, which upon mechanical fracture tends to produce a rougher fracture plane with more irregular surface features.

### 2.2. Linear Rheology

**Figure 2:**
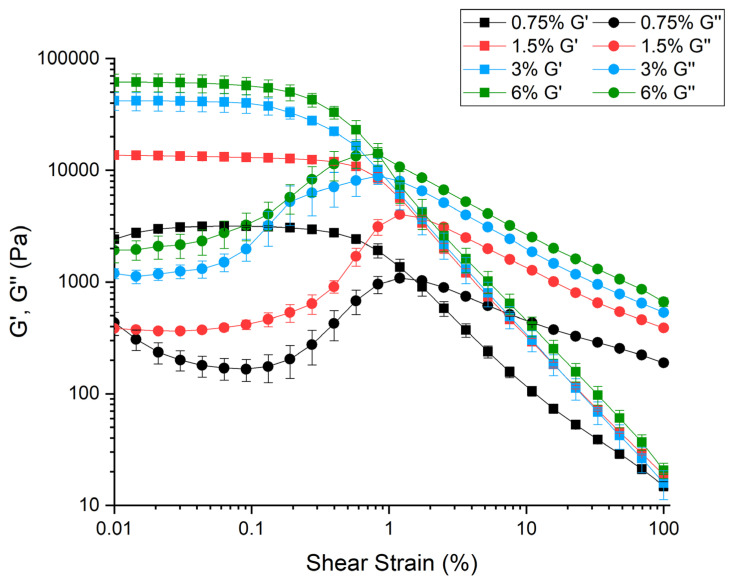
Amplitude sweep for different agar concentrations at a frequency of 10 rad/s. Black symbols and curves represent 0.75% agar, red 1.5%, blue 3%, and green 6% agar concentrations. Circles represent G′ modulus and squares G″ modulus.

Different microscopic surface morphologies suggest that at higher agar concentrations viscoelastic/solidification processes after surface disturbance outpace flow-driven smoothing. To check this, we have characterized viscoelastic properties of different agar gels both in linear and nonlinear shear strain domain. Increasing agar concentration led to a systematic increase in both storage modulus (G′) and loss modulus (G″) in the linear shear strain region as shown in Figure 2. The shape of the curves suggests gel network at all tested agar concentrations with G′ > G″. The 6% agar gel had the highest storage modulus, reaching 61,370 Pa (Table 1). The G′ increased 24-fold, whereas G″ increased 7-fold with increasing agar concentration from 0.75 to 6% agar. The loss factor, which shows how much energy on average is dissipated vs. stored, indicates that increasing agar concentration makes the network more elastic with less dissipation of energy. However, with increasing agar concentration the yield points as well as the flow points decreased significantly. A ductility index was defined as the strain interval between the yield and flow points, providing a measure of the material’s ability to sustain deformation after yielding. The ductility index decreased with increasing agar concentrations. This suggests more brittle behavior of agar with increased concentration. The 0.75% agar gel showed a distinct rheological behavior compared to the other samples. At low strain amplitudes, a decrease in G″ was observed, which was followed by a large increase until the flow point was reached. There was an overlap in G′ values observed in the non-linear part, which cannot be resolved with classical amplitude sweep; therefore, we have used LAOS measurements in the rest of the work.

### 2.3. Non-Linear Rheology

**Figure 3:**
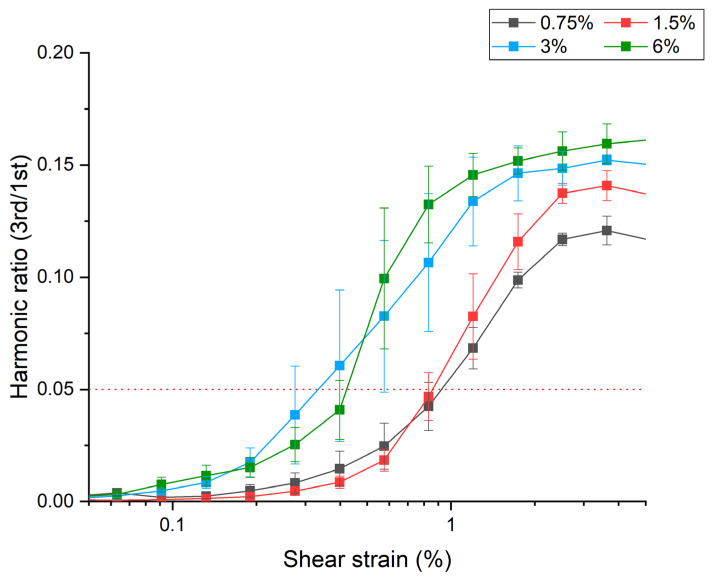
The 3rd/1st harmonic moduli ratio for different agar concentrations at 10 rad/s. Red dotted line is at value 0.05, which marks the transition into nonlinear range [29].

Different slopes of storage and loss moduli in the nonlinear shear strain regime indicate different agar breakdown mechanisms. To quantify the transition to nonlinear behavior, Fourier-transform (FT) rheology was applied to analyze the stress response. The stress signal was decomposed into its harmonic contributions, and the third-to-first harmonic intensity ratio (I_3_/I_1_) was used as a measure of nonlinear distortion. As shown in Figure 3, agar at higher concentrations enters the nonlinear regime at lower strain amplitudes. With a further increase in strain, a peak in the I_3_/I_1_ curve was observed, indicating the point of strongest nonlinear distortion of the material response. The maximum typically occurs at the transition from solid-like to yielding behavior, when the structure is highly stressed and nonlinear effects are strongest. At the point of the maximum of the I_3_/I_1_ curve, agar is neither fully intact nor fully broken. At this point bonds are breaking and reforming and the network is rearranging intensely. While the peak in I_3_/I_1_ occurs at a similar strain across agar concentrations, its magnitude increases with concentration, indicating stronger nonlinear distortion and a more densely connected, cooperatively responding network at higher agar concentrations.

**Figure 4:**
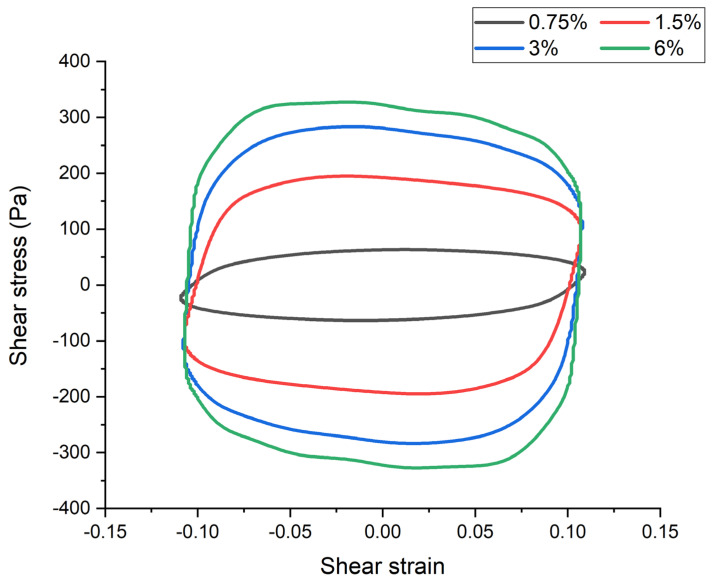
Lissajous–Bowditch stress versus strain curves (elastic response) for different agar concentrations at 11% shear strain and 10 rad/s.

The third-to-first harmonic intensity ratio (I_3_/I_1_) is a single scalar measure that quantifies the magnitude of nonlinear response. It is easy to compare across samples; however, two materials can have the same (I_3_/I_1_) ratio but very different mechanical behavior. To determine agar mechanical response within the strain cycle, Lissajous–Bowditch (LB) plots were constructed, showing stress vs. strain (elastic material response) or stress vs. strain rate (viscous behavior response) of the material. This allows a direct observation of shape distortion, intracycle behavior and symmetry/asymmetry response. In addition, LB plots allow one to determine the type of nonlinearity (elastic vs. viscous), intracycle processes (yielding, recovery, irreversible behavior), and structural mechanisms (smooth distortion and gradual rearrangement vs. sharp corners and brittle behavior). The intracycle elastic LB curves for different agar concentrations at 11% shear strain and 10 rad/s are given in Figure 4. At low agar concentration (0.75%) the LB curve is an elliptical, weakly tilted shape, which indicates little phase lag. There is only a slight distortion at high strain. This suggests a weak, flexible gel network with low density of junction zones. Chains can rearrange easily; the elastic response is dominant but soft. The material has mostly linear viscoelastic behavior with a late onset of nonlinearity at high strains. Deformation is gradual and continuous. The soft agar gel deforms smoothly before resisting. At high agar concentrations, LB curves changed markedly. The larger enclosed area reflects increased energy dissipation. At 6% agar the nearly square or box LB shape was observed, with sharp transitions at strain extremes. This is a strong departure from the ellipse, and it is consistent with a dense, highly connected brittle network with many junction zones and strong connectivity that constrains deformation. Stress rises sharply once the network becomes load-bearing. The material shows strong nonlinear elasticity, a sharp onset of strain stiffening and abrupt threshold-like mechanical response. The rigid gel resists deformation but breaks suddenly and strongly. During unloading (strain decreasing) stress slightly increased, indicating asymmetry and internal stress bias. This suggests that the network is rigid and slightly pre-stressed, so deformations in opposite directions are not equivalent. The LB shape change implies that higher agar concentrations make not just stronger gels but also have a qualitatively different deformation mechanism. In low-agar gels, there is weak progressive network deformation, whereas in high-agar gels, there is a collective, constraint-dominated response. The complete set of LB plots is given in Supplementary Materials.

**Figure 5:**
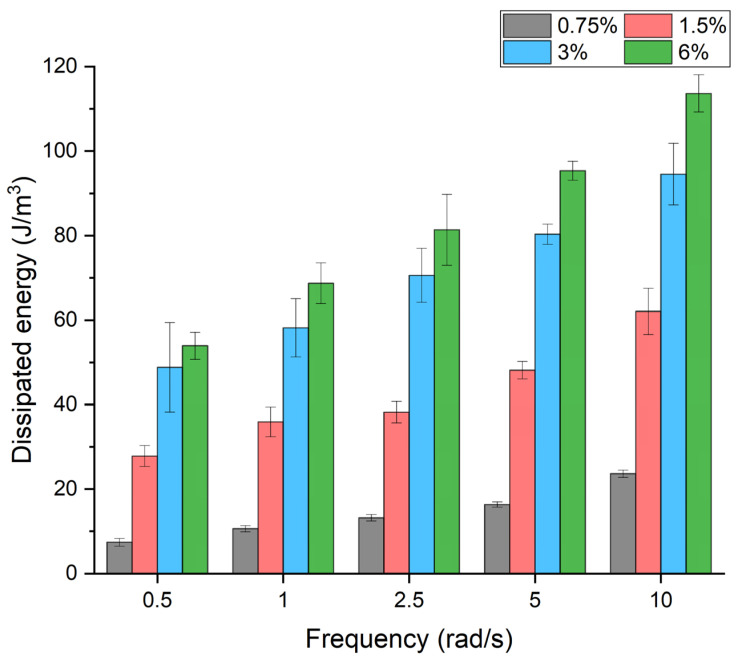
Dissipation energy for different agar concentrations and frequencies at 11% shear strain.

In a stress–strain LB plot the enclosed area of the loop equals energy dissipated per cycle. This is directly linked to viscous dissipation, internal friction, structural rearrangements, and irreversible deformation. The amount of dissipated energy at different frequencies during oscillatory straining in the nonlinear region at 11% shear strain is given in Figure 5. The soft 0.75% agar gel, having the lowest network connectivity, dissipates the least energy. The rate of dissipated energy increased with frequency of oscillations and agar concentrations.

**Figure 6:**
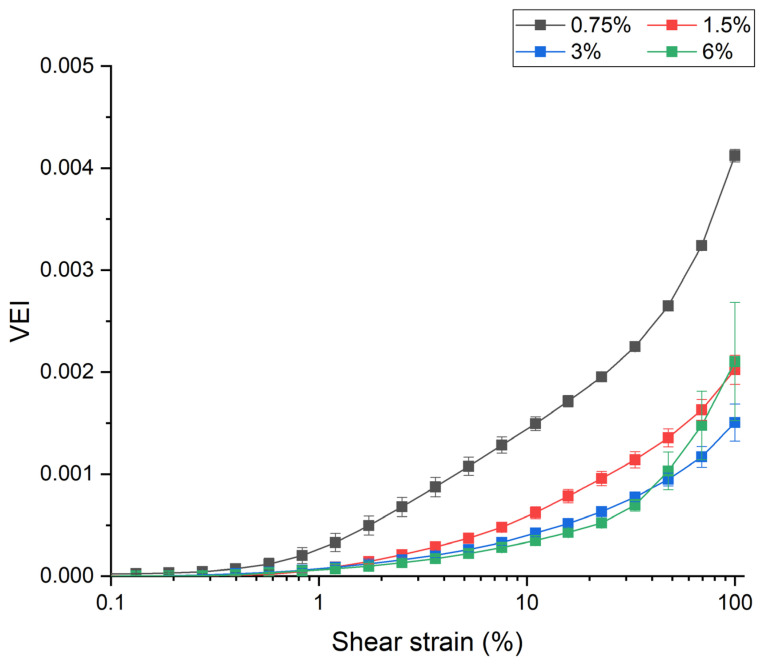
Viscoelasticity index, VEI, of different agar concentrations recorded at 10 rad/s.

To capture the balance between elastic and viscous contributions within a given LAOS strain–stress cycle, we define a viscoelasticity index (VEI) as the ratio of the minor to the major axis of the Lissajous–Bowditch curve. The VEI is an empirical descriptor and not a rigorous nonlinear rheological parameter. The VEI approaches 0 for purely elastic behavior and 1 for purely viscous behavior. As shown in Figure 6, agar behaves as an almost purely elastic material at low shear strains. With increasing strain, the elastic contribution decreases, yet agar remains predominantly elastic across all concentrations studied. At low agar concentration (0.75%), the VEI increases more markedly than at higher concentrations, indicating a greater shift toward viscous dissipation. Several distinct breakpoints appear in the VEI curve, at approximately 0.3, 0.7, and 30% shear strains, suggest different stages of structural rearrangement within the soft gel. Similar breakpoints are observed at higher agar concentrations, but they occur at corresponding higher shear strains.

**Figure 7:**
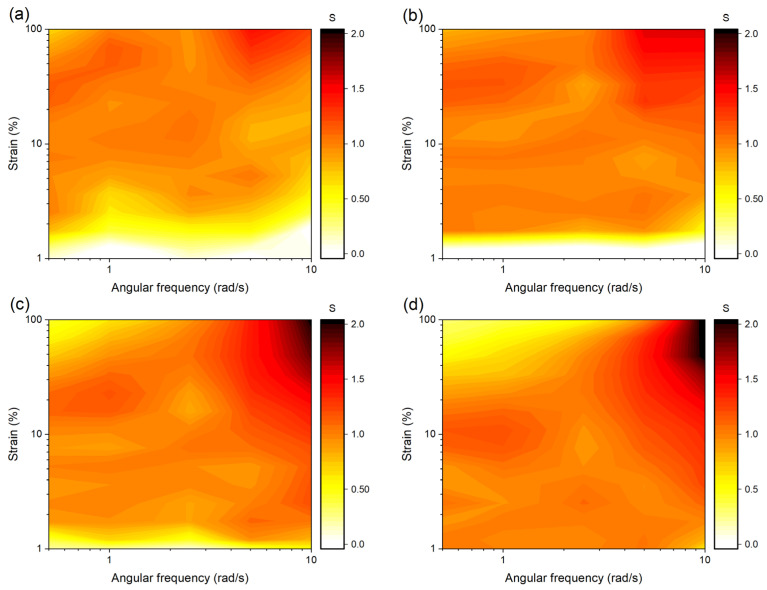
Fingerprint maps for strain-stiffening ratio S at different agar concentrations. (**a**) 0.75, (**b**) 1.5, (**c**) 3, and (**d**) 6% agar concentrations. Fingerprint maps were generated from representative measurements for each agar concentration.

The fingerprint maps of strain-stiffening at different agar concentrations are given in Figure 7. The strain-stiffening ratio S tells how a material’s stiffness changes as the material is deformed beyond the linear regime. If S > 0, material experiences strain stiffening; if S < 0, it experiences strain softening. When S > 1, elastic response increases more than twofold and the material experiences very high strain stiffening, often near structural limits. Here stress rises sharply; there is high nonlinear elasticity response and rapid activation of resisting network elements. The junctions are not deforming independently but respond collectively, which marks the emergence of load-bearing structures. Stress is concentrated in junction zones and there is an increased likelihood of sudden brittle-like failure and catastrophic yielding.

From Figure 7 one can observe that at low agar concentration (0.75%), material shows strain stiffening at all shear strains and frequencies in the nonlinear regime. More strain stiffening can be noticed in the upper right corner at higher frequencies and shear strains. The transition from the linear to nonlinear viscoelastic region can be observed at shear strain between 1 and 2%, where the S value was around zero. With increasing agar concentrations, one observes richer mechanical behavior. At low frequencies, S reached several local maxima, indicating large rearrangement of agar structure. This suggests that agar structure is undergoing stepwise rearrangements, with the processes likely including breaking/reforming of junction zones and sliding or reorganization of agarose helices. At low frequency, the system has time to rearrange and one can see multiple relaxation/restructuring events. The position of the local maximum S shifted toward lower shear strains with increasing agar concentrations. For example, at one rad/s, the maximum S was observed at 50, 30, 20, and 10% shear strain for agar concentrations 0.75, 1.5, 3, and 6%, respectively. This suggests that structural rearrangements become more constrained and occur at lower strain with higher agar concentrations. This is very informative and means that less deformation is needed to trigger structural rearrangement in denser gels. At higher agar concentrations the network is more tightly packed, junction zones are closer and more numerous, and even small strains quickly transmit stress through the network. This allows rearrangements earlier. It is important to note that S significantly decreased at low frequencies and high shear strains with increasing agar concentrations. The decrease in S at high strains in denser agars suggests that at large deformations the network is no longer rearranging efficiently. Instead, it is breaking and losing connectivity. In dense gels rearrangement pathways are limited, and instead of reorganizing the structure fails more abruptly.

The situation was very different at higher frequencies. Here strain stiffness ratio increased monotonically with increasing shear strain and reached S values above 2 at high agar concentrations in the upper right corner. In addition, one can observe an increased gradient of S radiating from the upper right corner with increasing agar concentrations. The mechanics of agar at high agar concentration (6%) and high shear strains with the increase in frequency is particularly rich. Here agar shows a dramatic increase in S going from low to high frequencies. At low frequencies, the elasticity increased by 50% during the intracycle, whereas at high frequencies, it increased by more than 200%. This is a very large change and indicates a qualitative transition in network mechanics, not just a gradual strengthening. At low frequency and S = 0.5, agar forms a moderately connected agar network where junction zones deform somewhat independently. At high frequency and S > 2, agar forms a highly interconnected agarose helix network, where junction zones respond cooperatively and collectively. Deformation activates load-bearing pathways; the network not only resists strain, but also strengthens under strain to the point of breaking. With an increase in frequency, the system evolves from a loose gel to a densely percolated elastic network with strong strain-induced reinforcement. At high frequencies the system approaches a brittle-like mechanical regime where gel becomes very stiff before failure.

**Figure 8:**
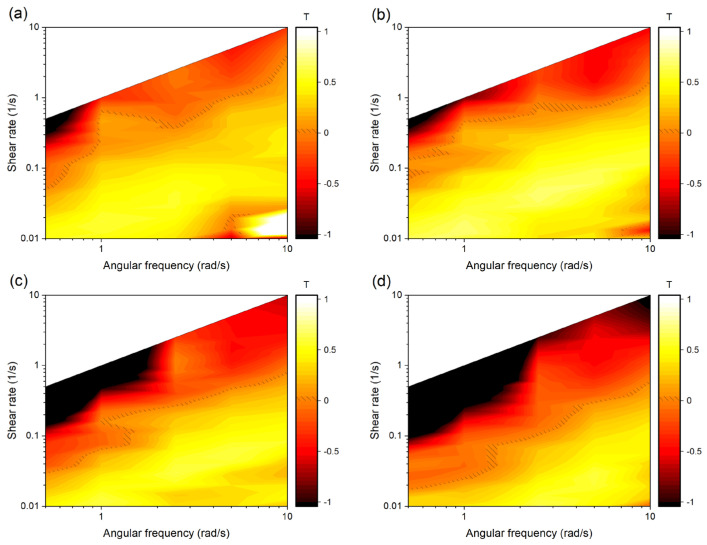
Fingerprint maps for shear-thickening ratio T at different agar concentrations. (**a**) 0.75, (**b**) 1.5, (**c**) 3, and (**d**) 6% agar concentration. No data were collected in the white upper left triangle. A value of T = 0 is indicated by dashed line and marks the transition between shear-thickening and shear-thinning behavior, corresponding to zero net nonlinear viscous response at the given strain rate. Fingerprint maps were generated from representative measurements for each agar concentration.

The shear-thickening fingerprint maps of different agar concentrations are given in Figure 8. One can observe a strong shear thickening dependence on agar concentration, shear rate and shear frequencies. At low agar concentration (0.75%) and low shear frequencies, agar shows shear thickening. This indicates nonlinear viscous response even at small deformations. Viscosity is changing within the cycle, and the response is not purely linear viscoelastic, suggesting microstructure rearrangements. Junction zones begin to reorganize under small deformation and local flow resistance increases during parts of the cycle. This is likely due to transient alignment of agarose helices and local densification of network regions. With the further increase in shear rate, T crosses the T = 0 line. The T = 0 represents the absence of net shear thickening or shear thinning at the corresponding strain rate. When crossing the T = 0 line the agar system transitions to dominant viscous weakening of the structure under deformation. At low agar concentrations, the network consists of sparse junction zones and a weakly connected percolated structure. Under high shear rates, these junctions are readily disrupted, and the polymer chains become increasingly aligned with the flow direction. As a result, the resistance to flow decreases, leading to pronounced shear-thinning behavior (T < 0).

Because the agar network undergoes structural collapse and helices disengage, flow alignment begins to dominate over network connectivity. As a result, the viscosity dissipation at high shear rates is much lower than at low deformation rates, and agar behaves more like a dilute polymer solution than a gel. When frequency increases one can observe that critical state of dynamic balance (T = 0) increases to higher shear rates. At higher frequencies, agar helices and water trapped within the network have less time to react to the movement. The reduction in cycle duration at higher frequencies limits the timeframe available for structural reorganization. Consequently, the agar network maintains its integrity over a greater portion of the oscillatory cycle, as the helices do not have sufficient time to fully disengage or undergo structural rearrangements. This necessitates a higher critical shear rate to overcome the kinetic barrier to network disruption, thereby delaying the transition from intracycle thickening to thinning.

With increasing agar concentration, the critical state of dynamic balance (T = 0) shifts to lower shear rates. While higher agar concentrations reinforce the network at low shear rates, they simultaneously introduce a structural fragility that promotes a catastrophic bifurcation at higher rates. This is evidenced by the shift of the T = 0 point toward lower shear rates as agar concentration increases. In these denser networks, the accumulation of stored elastic energy facilitates a more rapid transition to a flow-aligned state (T < 0). This suggests that the increased connectivity at higher agar concentration provides a higher ceiling for forced dissipation but also triggers a more dramatic structural collapse once the connectivity threshold is exceeded, leading to enhanced intracycle hysteresis and pronounced viscous nonlinearity. One might think a stronger gel stays intact longer, but because the dense network stores more elastic energy, it is under higher internal stress during deformation. The stored elastic energy contributes to network destabilization under increasing deformation. Once a critical shear rate is reached, the accumulated elastic energy exceeds the strength of the physical junction zones and the fracture resistance of the gel network, leading to rapid bond rupture, loss of network connectivity, and structural collapse. Consequently, the transition to T < 0 happens sooner because the breakdown is more explosive and efficient than the slow disengagement seen in thinner gels. These results imply a trade-off between structural robustness and structural fragility.

## 3. Discussion

In this work, we present a detailed analysis of the nonlinear rheological response of agar gels under shear deformation. Nonlinear rheology investigates the macroscopic stress–strain or stress–strain-rate behavior of materials to infer underlying microstructural dynamics. The central premise is that macroscopic nonlinearities observed arise from structural rearrangements within the material. Key molecular features governing agar dynamics such as bond rupture and reformation, junction zones, network connectivity, and chain alignment were not directly measured. Instead, they were inferred from rheological parameters, such as strain stiffening (S), shear thickening (T), Lissajous curve evolution, and higher harmonic responses (e.g., I_3_/I_1_). Importantly, nonlinear rheology extends, rather than replaces, linear viscoelasticity. In the limit of vanishing strain amplitude, the response becomes purely sinusoidal, higher harmonics disappear, and the system reduces to the linear regime. While linear measurements remain useful, most functional behavior of complex materials occurs outside this regime, making nonlinear characterization significantly more informative for practical applications.

This study demonstrates that increasing agar concentration produces not only quantitative changes in gel stiffness, as reflected in the increase in the storage modulus (G′), but also a qualitative transition in nonlinear deformation behavior that is not captured by linear viscoelasticity alone. The concentration-dependent increase in G′ is consistent with established understanding of agar systems, where higher polymer content promotes the formation of a denser junction-zone network based on agarose helix aggregation and physical crosslinking [2,4,16]. However, most previous studies have primarily focused on linear rheological descriptors and gel strength [4,7,13,16,23,24], which do not resolve how such networks reorganize and fail under large deformations. In contrast, the present nonlinear rheological analysis reveals that agar concentration governs the balance between structural rearrangement and structural constraint during deformation. At low concentrations, agar behaves as a weakly connected network capable of accommodating deformation through gradual rearrangement of junction zones and polymer chains. This is consistent with earlier descriptions of dilute agar gels as heterogeneous, loosely percolated networks with relatively high molecular mobility and delayed network failure [2,3,4,10,13,16]. In this regime, smooth fracture morphologies delayed the onset of nonlinearity, and a greater contribution of viscous dissipation reflects a deformation mechanism dominated by local rearrangement and partial recovery as also observed by microscopy.

The microscopy was used to qualitatively compare surface fracture morphology, brittleness, crack formation, and surface recovery following mechanical disruption. These features can be readily observed using light microscopy and provide complementary information regarding the mechanical response of agar gels of different concentrations [4,10,13]. The agar material at low concentrations exhibited an apparent resealing behavior following disturbance, characterized by recovery of continuity and partial restoration of its original shape. However, microscopy observations alone are insufficient to conclusively differentiate true network self-healing from other surface-related processes that can restore the original shape of the material [30]. The observed recovery may arise from surface water migration, capillary-driven closure of damaged regions, and the rearrangement of loosely connected polymer chains within the weak gel network, rather than from molecular-scale healing of the network itself [31,32].

At higher concentrations, the gel structure evolves toward a densely interconnected network, consistent with the classical picture of agarose helix bundle formation and increased junction-zone density reported in the literature [2,4,10]. The LAOS results show that this densification does not simply enhance elasticity; it fundamentally alters the deformation pathway. High-concentration gels exhibit earlier onset of nonlinear response, pronounced intracycle strain stiffening, strong harmonic distortion, and rough fracture morphologies, implying a transition to a cooperative, constraint-dominated response in which deformation is transmitted through a load-bearing network of interconnected junction zones.

A key finding of this study is that the characteristic strain marking the onset of structural reorganization shifts systematically to lower values with increasing agar concentration. Although previous studies have reported earlier yielding and greater brittleness in concentrated agar gels [4,12,16,25], the present results show that nonlinear effects emerge at progressively smaller deformations as concentration increases, as revealed by the LAOS metrics and intracycle analysis. Thus, despite their higher small-strain stiffness, denser agar networks begin to reorganize at lower strains. This behavior highlights a decoupling between linear stiffness and nonlinear deformability at high concentration in agar hydrogels.

Taken together, the results indicate that increasing agar concentration drives a transition in network mechanics that cannot be captured by linear viscoelasticity alone. While higher concentrations promote a denser and more interconnected junction-zone network consistent with established gelation models, nonlinear rheology reveals that this structural reinforcement is accompanied by a shift in deformation pathways. Specifically, the system evolves from a regime dominated by local rearrangement of weakly connected structures at low agar concentrations to one governed by cooperative, constraint-driven response and more abrupt failure under large deformation at high agar concentrations. This demonstrates that agar concentration affects not only the strength of the gel network, but also the mechanism by which it accommodates and dissipates mechanical stress.

## 4. Conclusions

In this study, linear and nonlinear rheology were combined to investigate the concentration-dependent mechanical behavior of agar hydrogels. While linear measurements captured the expected increase in gel stiffness with concentration, they did not describe how the network responds and fails under large deformation. Nonlinear rheology revealed that structural reorganization shifts systematically toward lower shear strain values with increasing agar concentration. These differences are reflected in nonlinear rheological metrics and supported by microscopic fracture observations. Overall, increasing agar concentration does not simply strengthen the gel; it fundamentally changes the deformation mechanism. LAOS reveals a transition from a rearrangement-dominated network at low agar concentrations to a constraint-dominated network at high agar concentrations that cannot be seen from linear rheology alone.

## 5. Materials and Methods

### 5.1. Agar Gel Preparation

Agar powder (Sigma-Aldrich, St. Louis, MO, USA) was weighed into a glass flask and dissolved in 50 mL of deionized water to obtain final concentrations of 0.75, 1.5, 3, and 6% (*w*/*v*). The agar used in this study was a standard Millipore microbiology-grade agar powder (Product No. 01916, SIAL; CAS: 9002-18-0). The product has pH 6.0–7.5, gel strength ≥ 800 g/cm^2^, gelling temperature 34–36 °C, and melting temperature 85–95 °C. The suspensions were heated and brought to boil in a microwave oven to ensure complete dissolution of the agar. Agar suspensions (50 mL) were heated in a microwave oven at 800 W for approximately 60 s until complete dissolution was achieved. During heating, the suspensions were manually mixed approximately three times to promote uniform heat distribution and minimize temperature gradients within the sample. The same heating protocol was applied to all agar concentrations throughout the study to ensure consistent gel preparation conditions. Following boiling, the solutions were maintained in a temperature-controlled water bath at 55 °C for 30 min to remove air bubbles formed during boiling. Next, 13 mL of each agar solution was poured into 90 mm diameter Petri dishes. This volume was selected to produce the minimum uniform gel thickness while ensuring complete coverage of the dish surface, even at the highest agar concentration (6%). The gels were allowed to cool and set at room temperature for at least 1 h prior to measurements. Circular samples, with a diameter of 25 mm and approximate thickness of 1.9 mm, were then cut using a custom-made 3D-printed mold and carefully transferred onto the rheometer measuring plate for analysis.

### 5.2. Determination of Agarose and Agaropectin Content

As the agar provider (Sigma-Aldrich, St. Louis, MO, USA) has not specified the mass content of agarose and agaropectin, we have determined the mass content of agarose and agaropectin by fractionation and gravimetric determination. The agarose and agaropectin contents of agar were determined using a modified dimethyl sulfoxide (DMSO) extraction method [33]. Agar powder was dispersed in DMSO at a concentration of 1% (*w*/*v*) and incubated at 70 °C for 2 h under continuous shaking. The suspension was subsequently centrifuged at 360× *g* for 20 min at 4 °C. The resulting supernatant was collected and mixed with an equal volume of distilled water. The mixture was then left at room temperature overnight to allow gel formation. The obtained agarose-rich gel was fragmented into small pieces and washed six times with distilled water to remove residual DMSO. Subsequently, the gel was washed twice with 95% (*v*/*v*) isopropanol to facilitate water removal and dehydration of the gel matrix. The purified agarose fraction was dried at 50 °C until constant weight was achieved. The agarose content was calculated gravimetrically from the dry mass of the recovered agarose fraction relative to the initial mass of agar. The agaropectin content was determined by difference according to: agarose (%) = (mass of recovered agarose/initial agar mass) × 100, and agaropectin (%) = 100 − agarose (%). All measurements were performed in quadruplicate (*n* = 4), and the results are reported as mean ± standard error in Table 2.

### 5.3. Rheology

All rheological measurements were carried out using an MCR 302 rheometer (Anton Paar, Graz, Austria). Large Amplitude Oscillatory Shear (LAOS) measurements were performed in oscillatory mode at angular frequencies of 0.5, 1, 2.5, 5, and 10 rad/s. The applied shear strain amplitude ranged from 0.01 to 100%, allowing characterization of both the linear and nonlinear viscoelastic response of the samples. The experiments were conducted using a parallel-plate geometry (PP25) with a plate diameter of 25 mm. The temperature was maintained at 20 °C. Prior to each measurement, a constant normal force of 1 N was applied to ensure reproducible sample loading, while the resulting gap, approximately 1.8 mm, was allowed to adjust accordingly. The measurement conditions were selected to ensure stable contact between the sample and the measuring surfaces while minimizing structural disturbance of the material. To reduce water evaporation during prolonged measurements, samples were enclosed using a custom-designed 3D-printed cover. This setup enabled stable measurement conditions over extended time periods and minimized potential artifacts associated with sample drying. All measurements were performed at least in triplicate to ensure reproducibility. The data obtained were analyzed after reaching steady oscillatory response, and the results are presented as mean values with the corresponding standard error.

### 5.4. Linear Viscoelastic Region Analysis

Within the linear viscoelastic regime, the storage modulus (G′) and loss modulus (G″) were measured and used to determine the yield and flow points. The yield point was defined as the strain at which G′ decreased by more than 5% from its initial plateau value. The flow point was identified as the strain at which a crossover between G′ and G″ occurred (G′ = G″). The loss factor tand was calculated as a ratio G″/G′ to indicate how much energy on average is dissipated vs. stored in the material under linear viscoelastic regime. A ductility index was defined as the strain interval between the yield and flow points (g_f_ − g_y_), providing a measure of the material’s ability to sustain deformation after yielding.

### 5.5. Nonlinear LAOS Analysis

In the nonlinear or large shear deformation regime, the stress waveform became non-sinusoidal and usually out of phase. The waveform stress signal obtained from the rheometer was analyzed by Fourier analysis using Python software (3.14.4). The odd harmonics (*n* = 1, 3, 5…) were used [34]. The intensity of third harmonics relative to the first (I_3_/I_1_) was calculated to determine the transition of agar gels to the nonlinear region [30,32]. To characterize the material’s physical response, Lissajous–Bowditch (LB) plots were constructed, depicting stress versus strain (elastic response) and stress versus strain rate (viscous response). The area enclosed by the elastic LB curve, obtained via integration, represents the energy dissipated during a single oscillation cycle [21,30,31,32]. The rate of energy dissipation at different frequencies was estimated by linear fit.

The LB curve shape contains important information about the material viscoelastic response. A viscoelasticity index (VEI) was defined as the ratio between the minor and major axes of the LB curve. To determine the index, we used a geometric approach, where the major axis was defined as the longest dimension of the Lissajous–Bowditch curve passing through its center, while the minor axis was defined as the perpendicular dimension passing through the same center point. The center of the LB curve was defined as the centroid of all sampled points (xi,yi). The major dimension was the maximum distance between any two points on the curve passing through the centroid, while the minor dimension was determined along the orthogonal direction. The ratio of these two axes yields the VEI. The value of 0 indicates an ideally elastic material, whereas a value of 1 corresponds to ideally viscous material. In the nonlinear regime, Lissajous curves are often distorted and non-elliptical, which may make axes mathematically ill-defined and VEI should therefore be treated as an empirical descriptor and not a rigorous nonlinear rheological parameter. The VEI provides a quantitative measure of the relative contributions of elastic and viscous behavior in the nonlinear region of shear deformation. The VEI and tanδ are related metrices, but not identical, and the difference becomes important in nonlinear regimes. In the linear viscoelastic regime, the LB curve is a perfect ellipse and its geometry is governed by the phase angle d. Since tanδ is a function of the phase angle δ, the relation is:

(1) $$VEI=sin\delta =\frac{tan\delta }{\sqrt{1+{tan}^{2}\delta }}$$

which means that VEI and tanδ are monotonically related, and in the linear viscoelastic regime, the VEI is directly related to the loss factor through the phase angle. To determine strain stiffening and thickening, the following relations have been used [35]:

(2) $$S=\frac{{G^{\prime }}_{L}-\;{G^{\prime }}_{M}}{{G^{\prime }}_{M}}\;$$

(3) $$T=\frac{{{ƞ}^{\prime }}_{L}-{{ƞ}^{\prime }}_{M}}{{{ƞ}^{\prime }}_{M}}\;$$

where S is strain-stiffening index and T is shear-thickening index. $G_{L}^{\prime }$ is minimum-strain modulus (slope near γ ≈ 0), $G_{M}^{\prime }$ is large-strain modulus (slope at maximum strain), $\eta_{L}^{\prime }$ is minimum-rate viscosity (near $\dot{\gamma }$ ≈ 0) and $\eta_{M}^{\prime }$ is maximum-rate viscosity (at highest $\dot{\gamma }$). In the linear viscoelastic regime of LAOS, the material response is proportional to deformation, so there is no distortion within the cycle intracycle and no change in material properties. The stress–strain curve and stress–rate curve are perfect ellipses with corresponding strain stiffening parameter S = 0 and shear thickening parameter T = 0. Material behaves like a linear spring and dashpot, no structure is being rearranged during oscillation, and response is fully reversible and proportional.

In the nonlinear viscoelastic regime of LAOS, the key change is that the material response is no longer proportional to deformation, so the stress response becomes intracycle-dependent (material properties change during a single oscillation). This is where parameters like S (strain stiffening) and T (shear thickening) become meaningful. Instead of a single modulus or viscosity value, we now have G′(γ) and η($\dot{\gamma }$), the stress waveform becomes non-sinusoidal, and LB curves become distorted and non-elliptical. If S > 0, the network becomes stiffer when stretched, and if S < 0, the network breaks or rearranges under strain. If T > 0, there is shear thickening and resistance increases with flow, and if T < 0, there is shear thinning and structure aligns or breaks down. In the nonlinear regime microstructure evolves during one cycle. During the cycle, bonds stretch, bonds partially break and reform, chains align and then collapse, junction zones deform cooperatively and the material is not in equilibrium [36]. For the construction of fingerprint maps, shear strain, angular frequency, and the S ratio were used as defining parameters for the strain-stiffening analysis. The fingerprint maps were generated from representative measurements rather than average datasets. For the shear-thickening analysis, the shear rate was first calculated (Equation (4)), after which shear rate, angular frequency, and the T ratio were used to construct contour maps.


(4) $$\dot{\gamma }=\gamma \omega \;$$

### 5.6. Microscopy

Agar gels were prepared following the same protocol as described for rheological measurements. After gelation, the upper surface of each sample was mechanically disrupted by gentle scraping to induce fracture to expose the internal microstructure. To ensure repeatability, the surface scraping procedure was performed in triplicate for each agar concentration. The same scraper and identical scraping protocol were used for all agar samples to minimize experimental variability. The resulting fragments were carefully transferred onto a glass microscope slide and lightly pressed to ensure sufficient contact and stability during imaging. No coverslip was applied, and no additional hydration or drying steps were performed prior to observation. Microscopic analysis was carried out using an Axio Observer microscope (Zeiss, Oberkochen, Germany) equipped with differential interference contrast (DIC) optics at 10× magnification. Images were adjusted for brightness and contrast using ImageJ (1.54m), without further image processing.

## Figures and Tables

**Table 1:** Viscoelastic properties of different agars in the linear shear strain region.

	0.75%	1.5%	3%	6%
G′ (Pa)	2554 ± 272	13,460 ± 360	42,660 ± 5860	61,370 ± 11,280
G″ (Pa)	280 ± 22	990 ± 6	1158 ± 64	1990 ± 230
Loss factor (tanδ)	0.11	0.07	0.03	0.03
Yield point (%)	0.30 ± 0.03	0.23 ± 0.08	0.065 ± 0.007	0.083 ± 0.021
Flow point (%)	1.62 ± 0.2	1.7 ± 0.3	0.66 ± 0.15	0.54 ± 0.02
Ductility index Δγ	1.32	1.47	0.60	0.46

**Table 2:** Content of agarose and agaropectin in agar powder.

	Content (%)
Agarose	71 ± 4
Agaropectin	29 ± 4

## Data Availability

The original contributions presented in this study are included in the article/Supplementary Materials. Further inquiries can be directed to the corresponding author.

## Supplementary Materials

The following supporting information can be downloaded at: https://www.mdpi.com/article/10.3390/gels12070603/s1, Figure S1: Lissajous Bowditch stress versus strain plots (elastic response) for 0.75% agar. Figure S2: Lissajous Bowditch stress versus strain plots (elastic response) for 1.5% agar. Figure S3: Lissajous Bowditch stress versus strain plots (elastic response) for 3.0% agar. Figure S4: Lissajous Bowditch stress versus strain plots (elastic response) for 6.0% agar. Figure S5: Lissajous Bowditch stress versus shear rate plots (viscous response) for 0.75% agar. Figure S6: Lissajous Bowditch stress versus shear rate plots (viscous response) for 1.5% agar. Figure S7: Lissajous Bowditch stress versus shear rate plots (viscous response) for 3.0% agar. Figure S8: Lissajous Bowditch stress versus shear rate plots (viscous response) for 6.0% agar.
