# Supplementary material for: Concentration-Dependent Nonlinear Rheology of Agar Hydrogels
Source: Gels. 2026 Jul 7;12(7):603. doi: 10.3390/gels12070603 (PMC13408723; doi:10.3390/gels12070603)

Supplementary material

Figure S1: Lissajous Bowditch stress versus strain plots (elastic response) for 0.75 % agar.

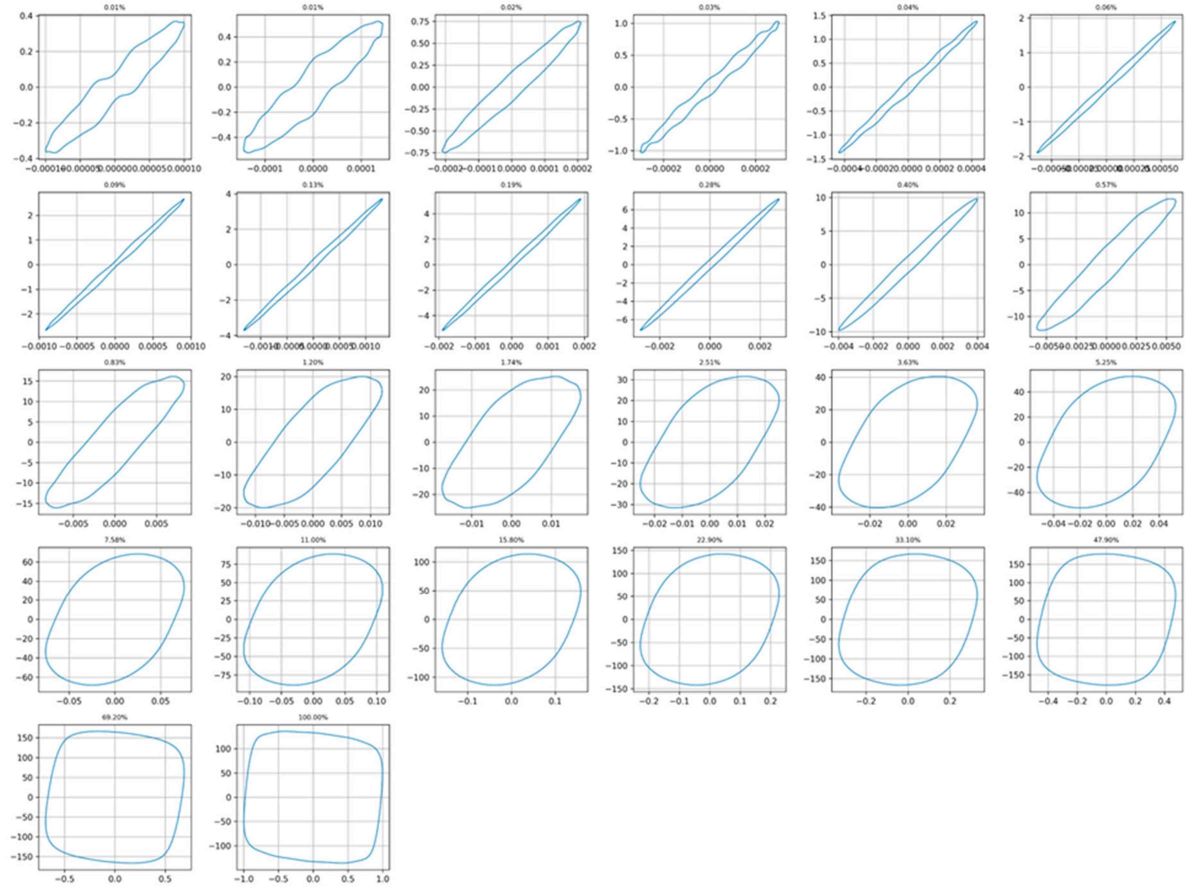

Figure S2: Lissajous Bowditch stress versus strain plots (elastic response) for 1.5 % agar.

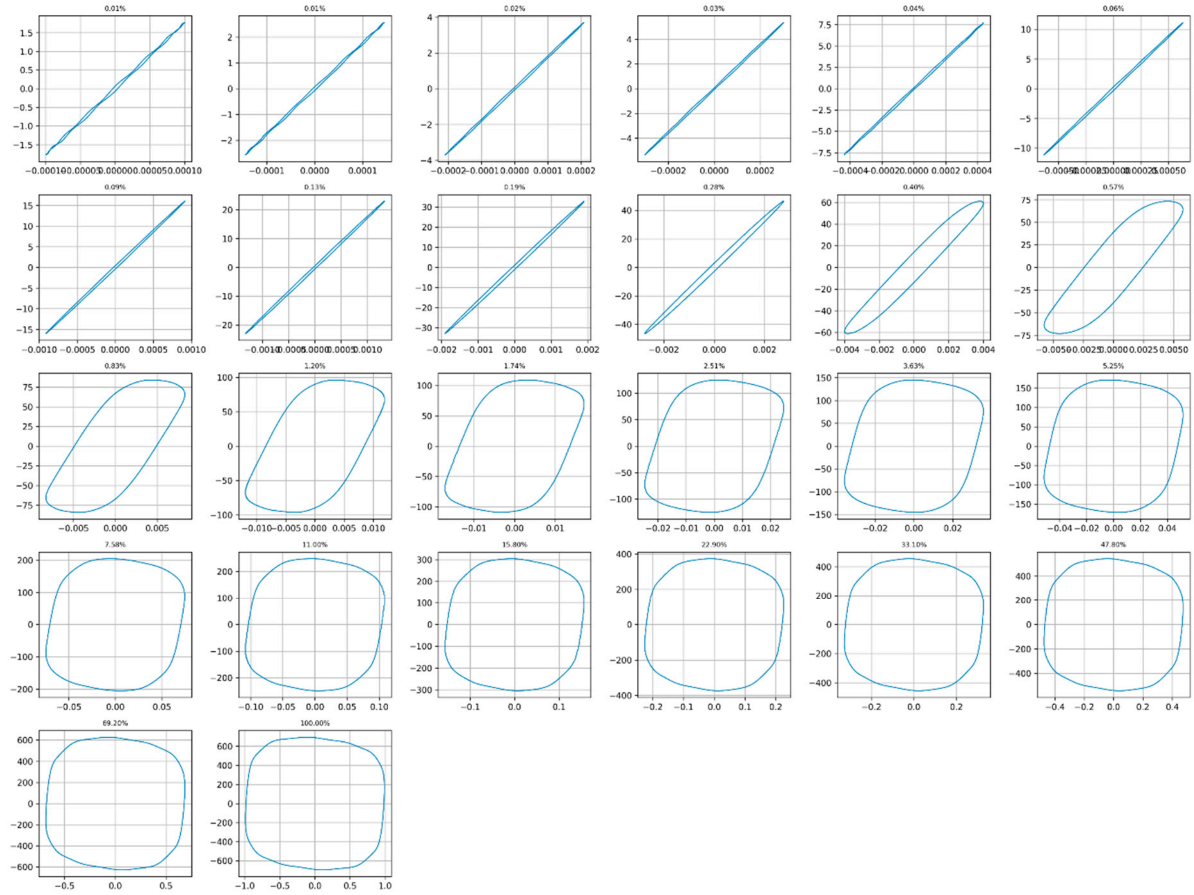

Figure S3: Lissajous Bowditch stress versus strain plots (elastic response) for 3.0 % agar.

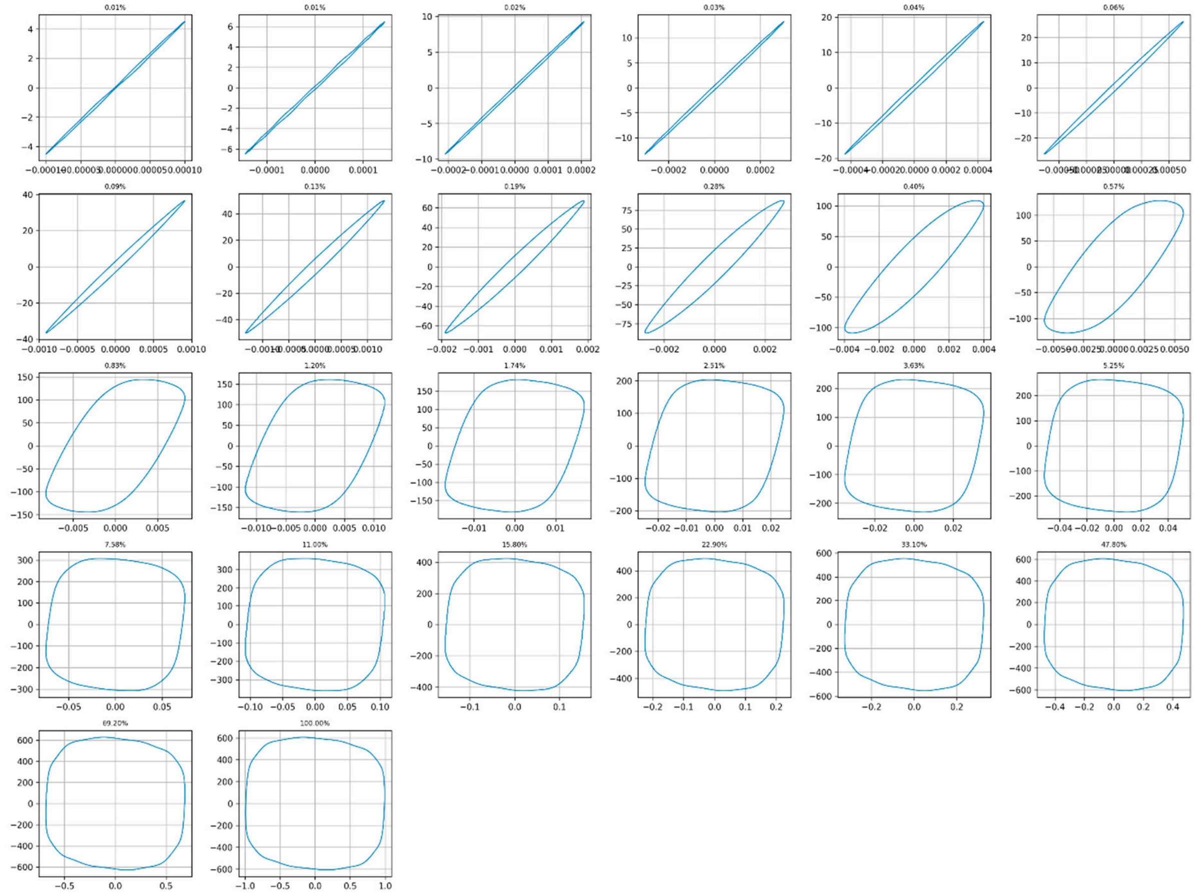

Figure S4: Lissajous Bowditch stress versus strain plots (elastic response) for 6.0 % agar.

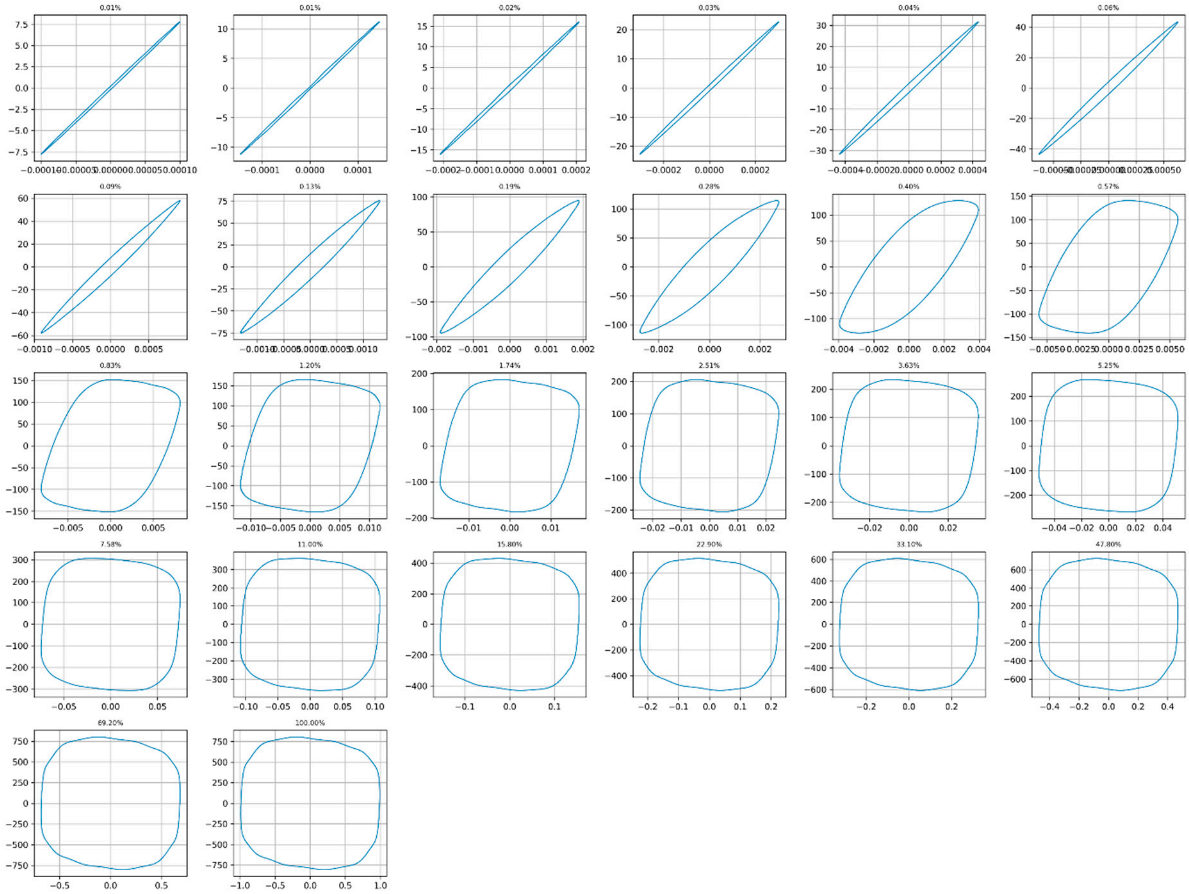

Figure S5: Lissajous Bowditch stress versus shear rate plots (viscous response) for 0.75 % agar.

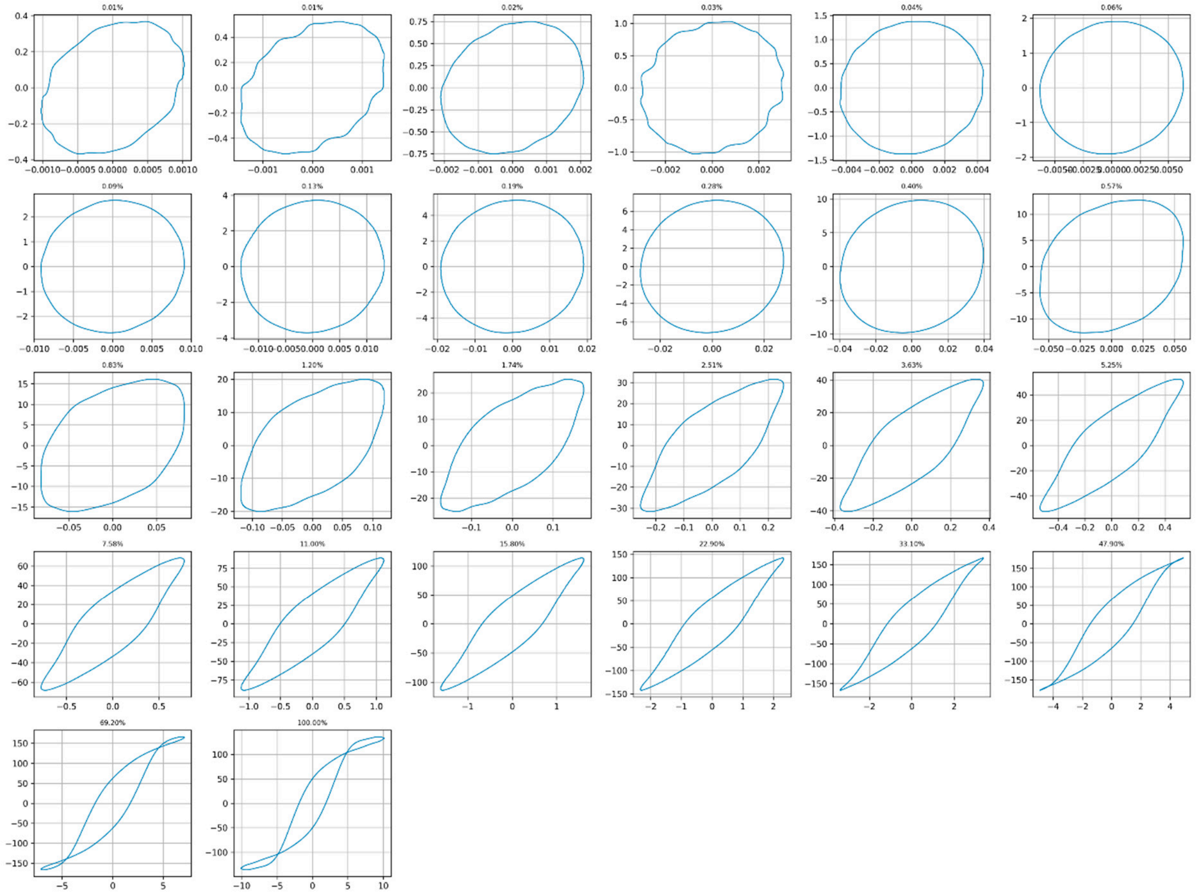

Figure S6: Lissajous Bowditch stress versus shear rate plots (viscous response) for 1.5 % agar.

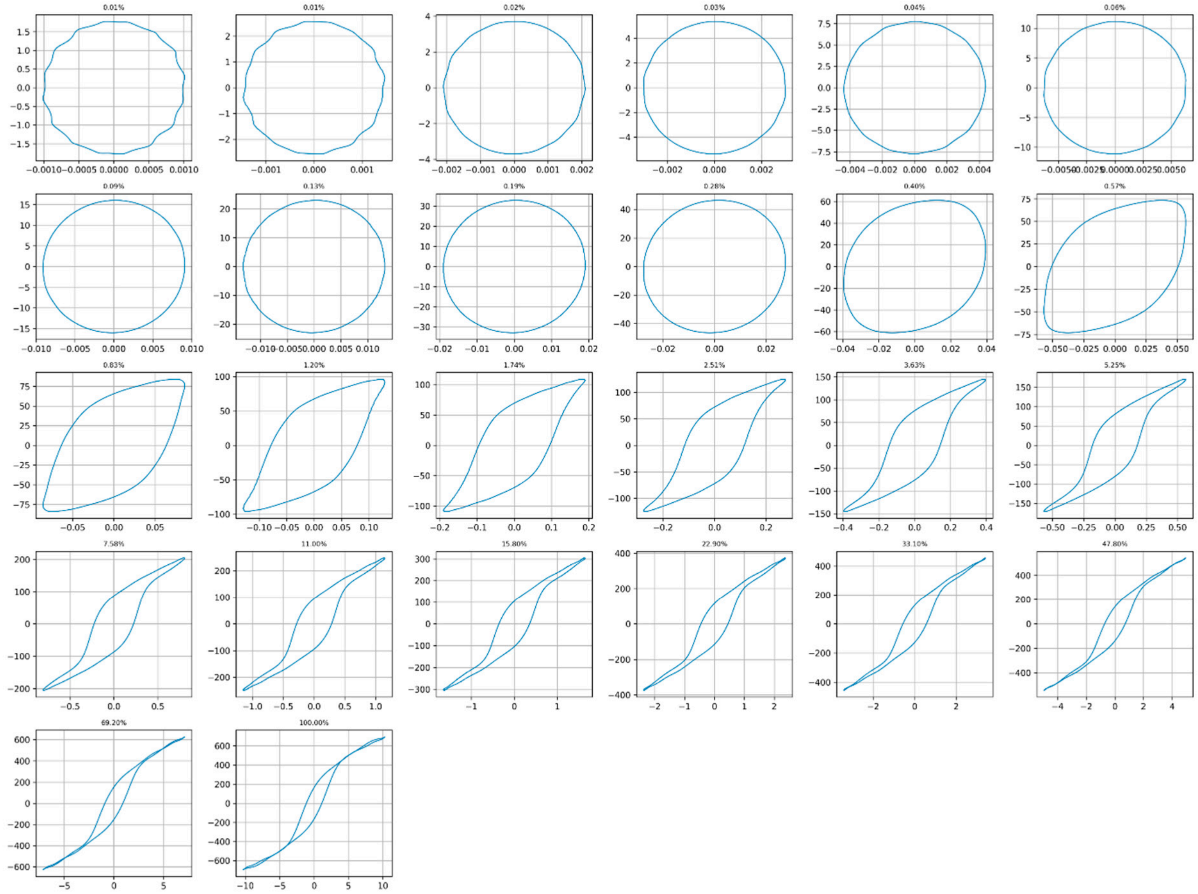

Figure S7: Lissajous Bowditch stress versus shear rate plots (viscous response) for 3.0 % agar.

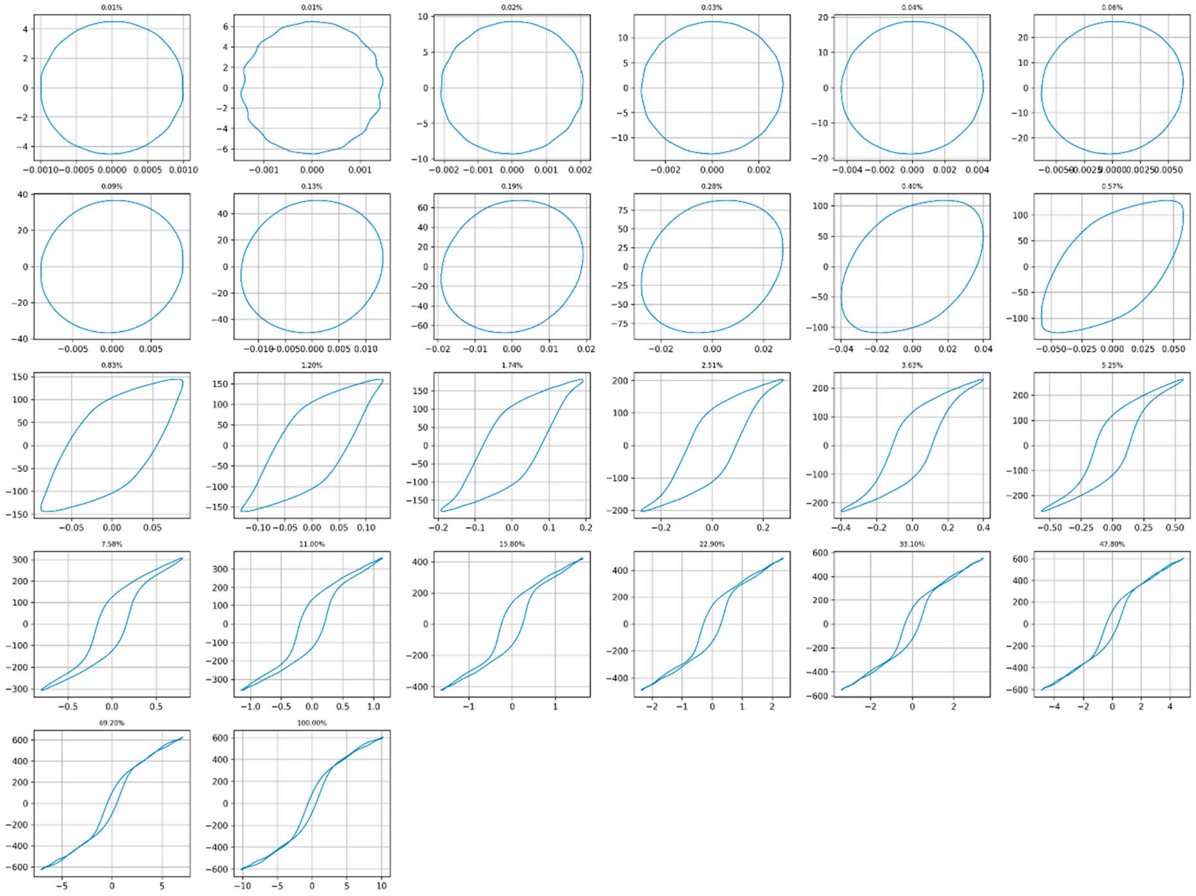

Figure S8: Lissajous Bowditch stress versus shear rate plots (viscous response) for 6.0 % agar.

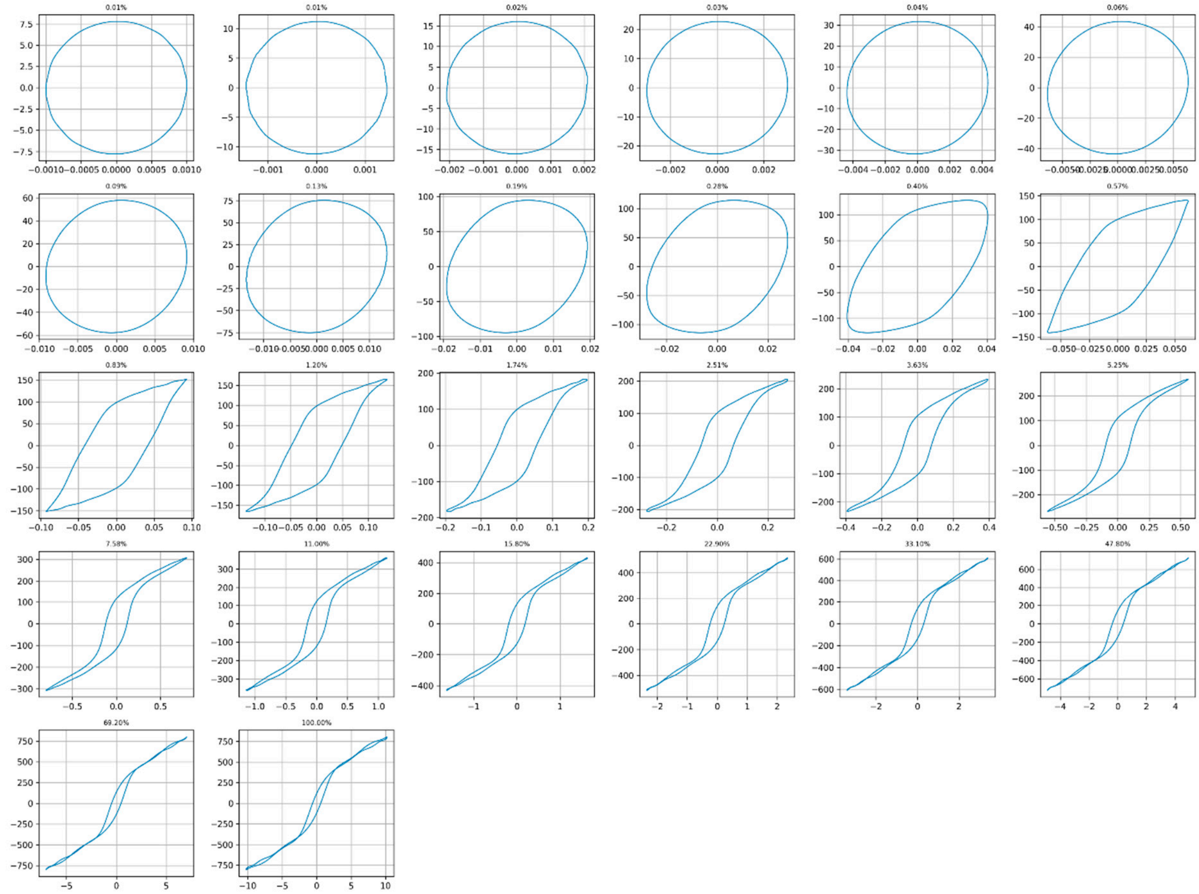

Supplement: Supplementary file 1 [file gels-12-00603-s001.zip › gels-4363208-supplementary.pdf]
